# Determining the need for additional training among hospital infection-control workforce – results from a multicentric survey within the multiresistance network of southern Lower Saxony (MRNS), Germany

**DOI:** 10.3205/dgkh000409

**Published:** 2022-04-11

**Authors:** Felix Barre, Hani Kaba, Isabella Dresselhaus, Eckart Mayr, Michelle Voigt, Reiner Schaumann, Marie-Luise Dierks, Simone Scheithauer

**Affiliations:** 1 Institute of Infection Control and Infectious Diseases, University Medicine Göttingen, Georg August University of Göttingen, Göttingen, Germany; 2 Health department for the city and the district of Göttingen, Göttingen, Germany; 3 Institute for Epidemiology, Social Medicine and Health System Research, Hannover Medical School, Hannover, Germany

**Keywords:** hygiene consultation, infection prevention, infection control, multi-drug resistant organisms, knowledge transfer, infection prevention

## Abstract

Infection-control nurses (ICN) and infection-control link physicians (ICLP) are both responsible for infection prevention practices in hospitals. However, their topic-specific education levels and extent of engagement in infection-control issues are diverse, creating potential needs for additional training. We aimed at determining the potential need for additional training in infection-control among ICN, ICLP and medical Chief Executive Officers (CEO) in hospitals of the Multiresistance Network of southern Lower Saxony (MRNS), via structured surveys (n=48; 55.1%). Our data suggest that the majority of ICN as well as ICLP have unmet needs for consultation and training on the topics of hospital hygiene and infection control. We observed a higher need for advice/additional information among ICLP than among ICN, e.g., concerning outbreaks (p=0.032), multidrug resistance (p=0.005) or antimicrobial stewardship (p=0.020). Therefore, future training programs might require targeting workforce-specific topics as part of their curricula. Furthermore, the improvement of the knowledge of ICN and ICLP for the implementation of infection control could contribute to improved prevention of the transmission of infectious diseases.

## Abstract (de)

Hygienefachkräfte (HFK) und Hygienebeauftragte Ärzte (HBÄ) sind beide für die Infektionsprävention in Krankenhäusern verantwortlich. Ihr themenspezifischer Ausbildungsstand und das Ausmaß ihres Engagements in Fragen der Infektionskontrolle sind jedoch unterschiedlich, was zu einem potenziellen Bedarf an weiterer, unterschiedlicher Beratung führt. Unser Ziel war es, den potenziellen Beratungsbedarf im Bereich der Infektionskontrolle bei HFK, HBÄ und ärztlichen Geschäftsführern (CEO) in den Krankenhäusern des Multiresistenznetzwerks Südniedersachsen (MRNS) mittels strukturierter Umfragen zu ermitteln (n=48; 55,1%). Unsere Daten deuten darauf hin, dass sowohl die Mehrheit der HFK als auch der HBÄ einen ungedeckten Bedarf an Beratung sowie an Schulungen zu den Themen Krankenhaushygiene und Infektionskontrolle haben. Wir beobachteten bei den HBÄ einen höheren Bedarf an Beratung/zusätzlichen Informationen als bei den HFK, z. B. in Bezug auf Ausbrüche (p=0,032), Multiresistenz (p=0,005) bzw. Antibiotic Stewardship (p=0,020). Künftige Schulungsprogramme sollten daher möglicherweise auf berufsspezifische Themen abzielen und diese in ihre Lehrpläne aufnehmen. Darüber hinaus könnte die Verbesserung der Kenntnisse von HFK und HBÄ bei der täglichen Arbeit zu einer verbesserten Prävention der Übertragung von Erregern und der Entstehung von Infektionskrankheiten beitragen.

## Introduction

Appropriate infection control staffing, which includes adequate staffing levels and sufficiently qualified personnel, is the cornerstone to prevent transmissions of multi-drug resistant organisms (MDRO) and health care-associated infections (HCAI) [1], [2]. Infection-control nurses (ICN) are the backbone of hospital hygiene management. Especially in smaller hospitals, a physician specialized in infection control is not or not always on site [3]. Katzmann et al. [4] found that regular interprofessional case discussions can transfer the latest knowledge about infectious diseases into clinical medical care, while Mangal et al. [5] also underlined the usefulness of knowledge acquisition for infection prevention of family members who must perform hygiene-related activities for relatives in the home environment. Chu et al. [6] used the example of the COVID-19 pandemic to show that knowledge of preventing infection should also serve as a basis for decision-making in politics. Specialist clinics are legally obliged to employ infection control link physicians (ICLP). They do not have specialized expertise in infection prevention, but they are required to attend a one-week basic course and participate in regular updates. ICLP are responsible for the implementation of the infection prevention and control measures at their clinics. In addition, in some countries, e.g., Germany, the hospital chief executive officers (CEO) are generally responsible for infection-control tasks. These health care staff groups are trained in infection control issues. Furthermore, they are involved in regular instruction and management issues and are obligated to take part in meetings and congresses. Additional information focusing on the latest knowledge about infectious diseases may be needed. Thus, our study aimed at investigating the potential need for additional instruction, information or consultation on infection prevention and control issues among infection-control staffing in terms of their daily work.

## Methods

The research questions were “Do ICN, ICLP and CEO have a self-perceived need for additional instruction, information and consultation on infection-control issues in their daily work?” and “What are the relevant issues, problem areas and weak points with regard to information and consultation on preventing infection?” In order to answer these questions, we performed a structured self-perceived needs survey. Thirteen clinics of the regional bacterial-resistance network in southern Lower Saxony (Germany) (https://gesundheitsregiongoettingen.de/mrenetzwerk/) were asked to participate in the study by completing a quantitative questionnaire. Informed consent was obtained; approval was given by the local ethics committee (33/8/19). The respondents were asked to choose

1. potential topics with need for consultation in daily routine,
2. the time point at which the consultation request should be answered,
3. the preferred means of communication, and
4. the preferred consulting institution.

**Figure 1:**
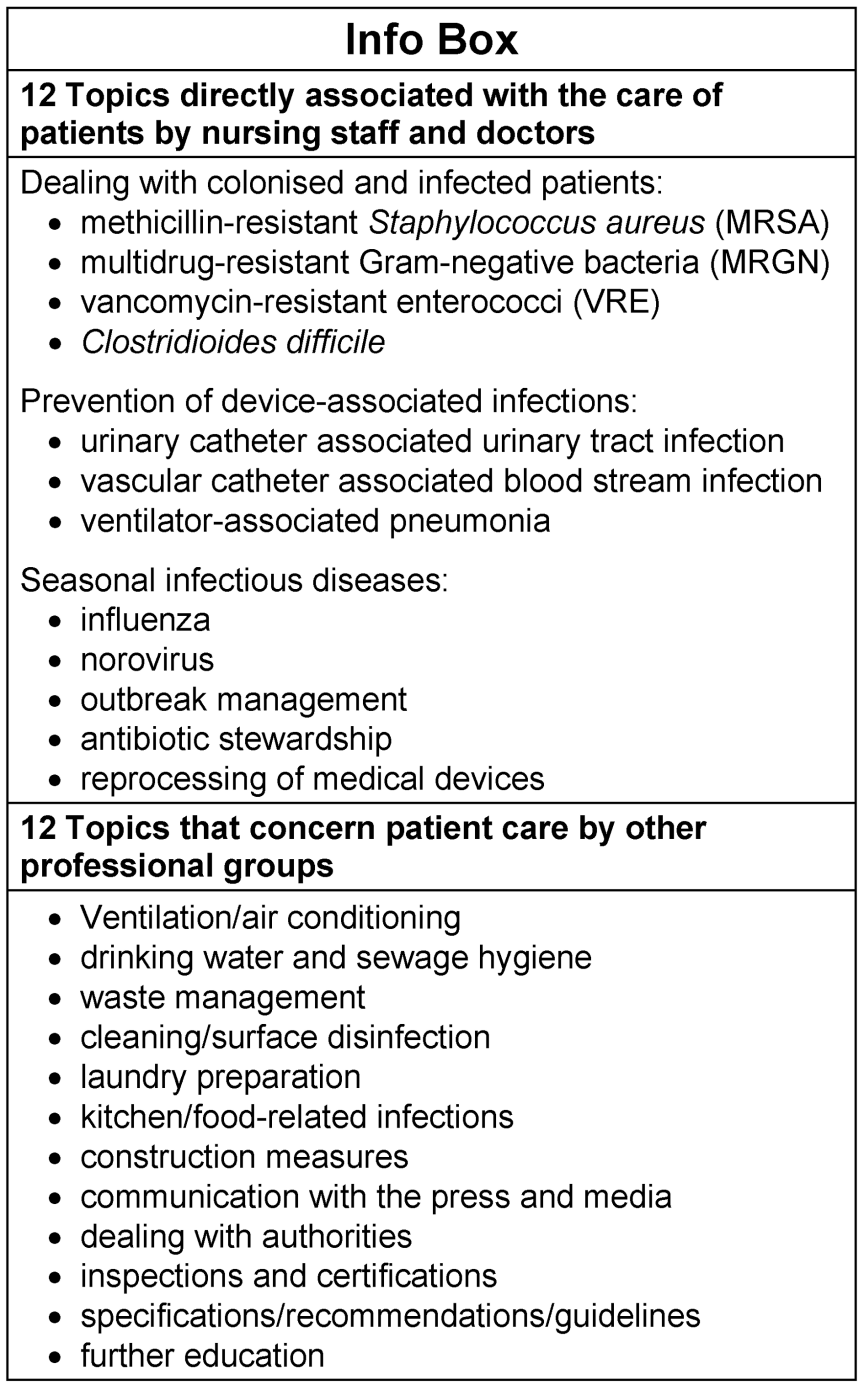
24 specialist topics of hospital hygiene available as possible consultation topics in the structured questionnaire

The 24 topics are listed in the info box in Figure 1 (Fig. 1). 

The different number of topics among the three healthcare worker groups was determined using the Mann-Whitney U-test. The difference between the desired means of communication and sense of urgency (time point of consultation) was determined using the Chi-squared test.

The surveys were sent out on September 30^th^, 2019 and were completed and returned by November 30^th^, 2019.

## Results

A total of 87 healthcare workers were addressed (ICN 23, ICLP 51, CEO 13) and the response rates were 69.6% (16 of 23), 52.9% (27 of 51) and 38.5% (5 of 13), respectively. The mean number of topics of interest differed between the groups, with the highest number of relevant topics given by the ICLP (11.9; SD: 5.3), followed by the ICN (7.9; SD: 6.4) and lastly the CEO (6.8; SD: 6.6). The difference between ICN and ICLP was statistically significant (p<0.05). A comparison with the CEO was not made because of the small size of the group. For distribution of topics, see Table 1 (Tab. 1).

The majority of respondents – 56.7% in the ICN group, 85.8% in the ICLP group and 100% in the CEO group – would like information and consultation on all survey topics within at least 24 hours after the initial request. In comparison to the other groups, a higher percentage of ICLP (42.9%) wished to receive the information immediately after the request. Table 2 (Tab. 2) and Table 3 (Tab. 3) illustrate the prioritization of the consultation requests by ICLP and ICN depending on topics, importance and sense of urgency. 

**Figure 2:**
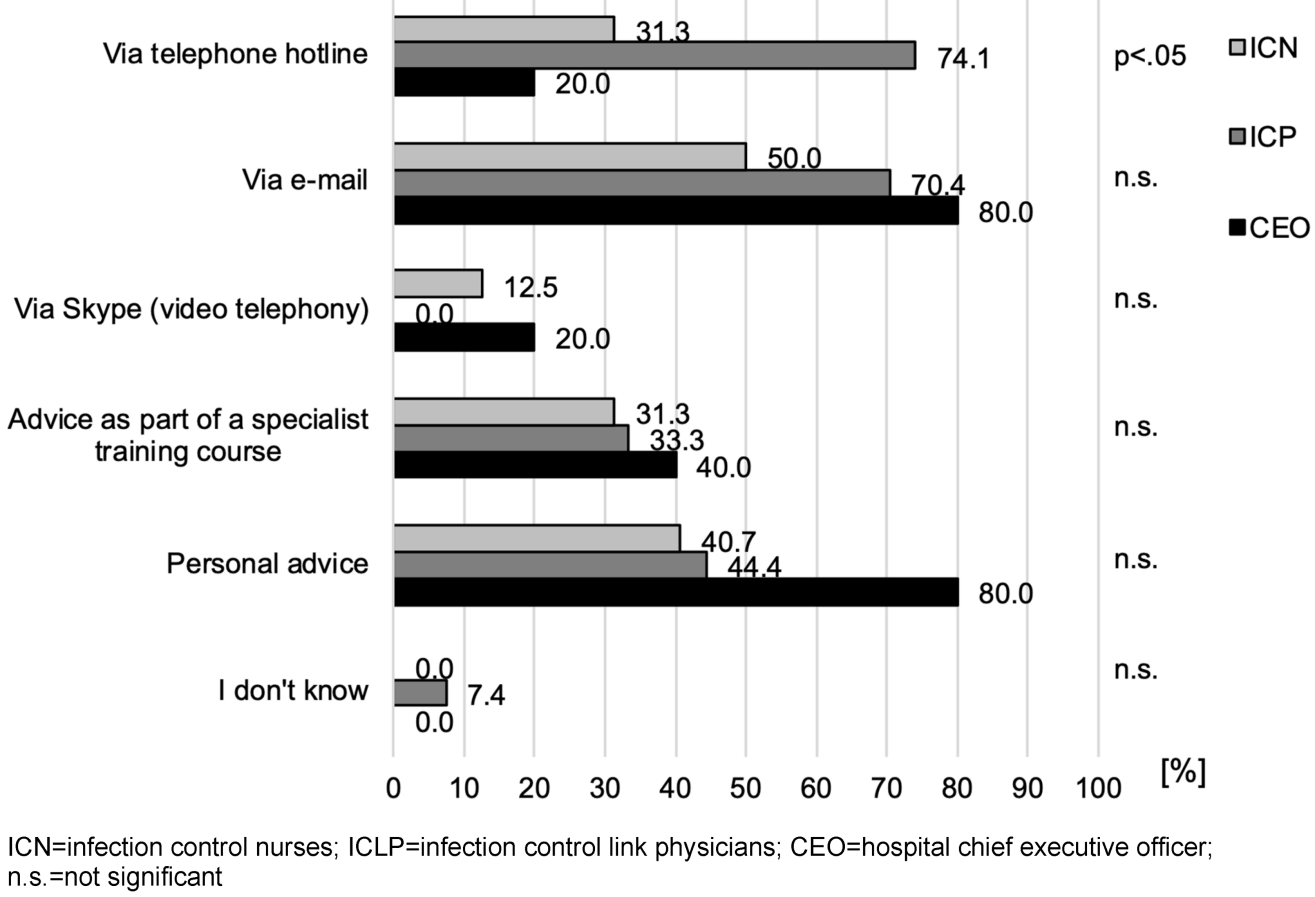
Forms of consultation and media (multiple answers possible), stated in % of those surveyed

The preferred means of communication differed between the three healthcare worker groups. The ICN and CEO preferred e-mail and personal consultation, whereas ICLP favoured consultation by telephone and e-mail. The only statistically significant difference between the ICN and ICLP groups was observed for the preference for consultation by telephone hotline (p<0.05). No differences were observed regarding the preferences of the CEO with either group. For preference distributions, see Figure 2 (Fig. 2).

ICNs preferred university institutes (23.9%), other clinics (20.9%) and public health services (17.9%) for acquiring expertise and consultation on topics associated with the care of patients by nursing staff and doctors. In the ICLP group, the vast majority would like to receive consultation from university institutes (69.3%). The CEO group largely preferred consultation by public health services (50%), followed by university institutes (38.9%) (see Table 4 (Tab. 4)).

## Discussion

Although the ICN and ICLP are qualified in the field of hospital hygiene, there was a need for additional information on almost all the topics offered in the survey. The fact that the ICLP had a higher need for additional information/consulting compared to the ICN, as measured in terms of the number of topics, can be explained by the high dynamic of the professional field and the larger area of responsibility. This is also indicated by the ICLP’s sense of urgency for information/consulting and the preferred advisory institution (university). Humphreys [7], who investigated telephone consulting by a clinical microbiology department from 2008 to 2018, found that consultation was most often related to treatment and diagnosis rather than infection prevention and control. More than half of the callers were doctors, while fewer callers were nurses dealing with IPC matters.

The ICN group, on the other hand, preferred an increased exchange with other clinics. Since professional exchange of knowledge is limited within many smaller clinics, as they often have a single ICN, an exchange of practical implementation with external experts would probably be very useful. Networks help to identify the existing structures in the hospitals, point out gaps, and the communication possibilities between the facilities [8]. The most preferred means of communication by the ICN and ICLP were telephone and e-mail. Complex cases or issues can be resolved quickly by telephone, while e-mails enable time-independent inquiries and sending documents.

The topics most cited by both professional groups concerned dealing with patients with the most common pathogens MRSA, MRGN, VRE and Clostridioides [9]. One third of the participants in the survey sample expressed their wish for further training. The Lower Saxony Ordinance on Hygiene and Infection Prevention in Medical Institutions [10] stipulates that every two years, ICN and ICLP must participate in regular educational updates on hygiene, infection prevention and the use of antibiotics. It is possible that this does not cover the actual needs of those responsible for hygiene, so that the organization of needs-based training can be useful.

The CEOs’ need for consultation cannot be clearly determined due to the small number of participants. Further investigations are necessary on this issue.

It is worth mentioning that our sample was not randomly selected, but rather restricted to 13 centers of the MRNS network. In our explorative approach, we anonymously targeted all ICN and ICLP professionals who were registered at these centers, with a response proportion of >50%. Still, our sample might not be suited to generalizing our findings to the broader (national or international) population of infection-control workforce and are limited to the regional level under observation. Nevertheless, these findings provide a valuable insight into potential group-specific needs of infection control professionals, presenting a basis for future hypothesis-driven interventions.

The written survey was conducted before the start of the COVID-19 pandemic. It can be assumed that the need for consultation has increased due to constant new knowledge on the subject area.

Overall, a great need for information/consulting was identified, which should be organized, provided and evaluated promptly in the context of the current COVID-19 pandemic.

## Conclusions

The aim of this study was to determine the need for additional information/consulting on infection prevention and control by specific occupational groups. Clinics of the MRE network in southern Lower Saxony participated in this study.

Our data suggest that the majority of both ICN and ICLP have an unmet need for consultation, information, and training on the topic of prevention and infection control. The desired consultation topics are: patients colonized or infected with multi-drug resistant gram-negative bacteria, vancomycin-resistant enterococci, outbreak management, antibiotic stewardship, construction measures, inspections and certifications, and dealing with legal requirements and guidelines. Improving the knowledge of ICN and ICLP may contribute to more effective prevention of the transmission of multi-drug resistant bacteria and other pathogens, and the prevention of health-care-associated infections [8]. Appropriate consultation options should therefore be made available as soon as possible.

## Notes

### Competing interests

The authors declare that they have no competing interests.

### Funding

The authors received no funding.

### Acknowledgement

The authors are grateful for the strong commitment of the participants in the MRE network southern Lower Saxony. Without their support, this study would not have been possible.

## Figures and Tables

**Table 1:**
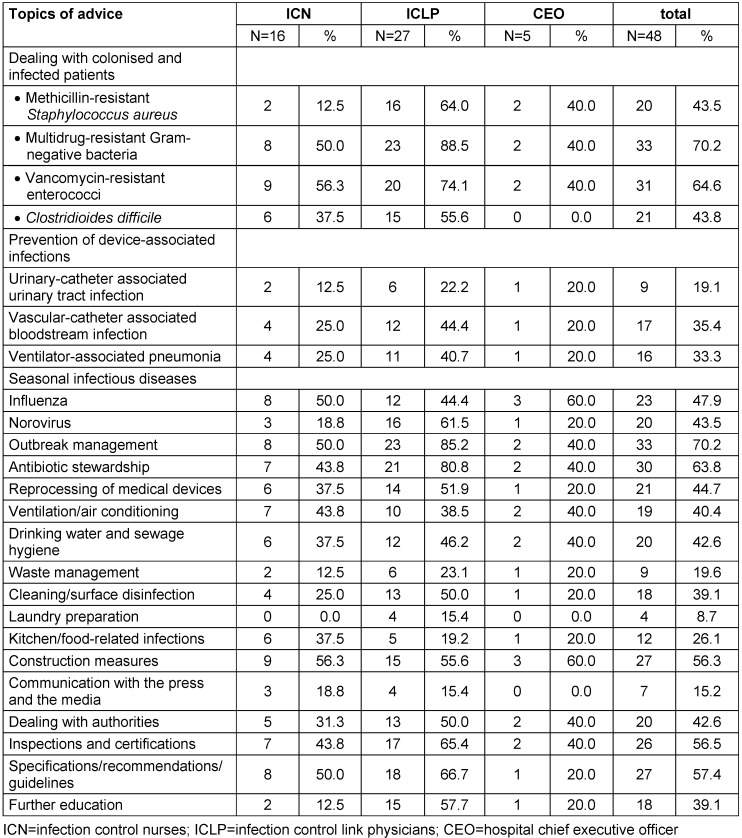
Topic-specific need for consultation “On which topic do you have any questions or would you seek consultation?”

**Table 2:**
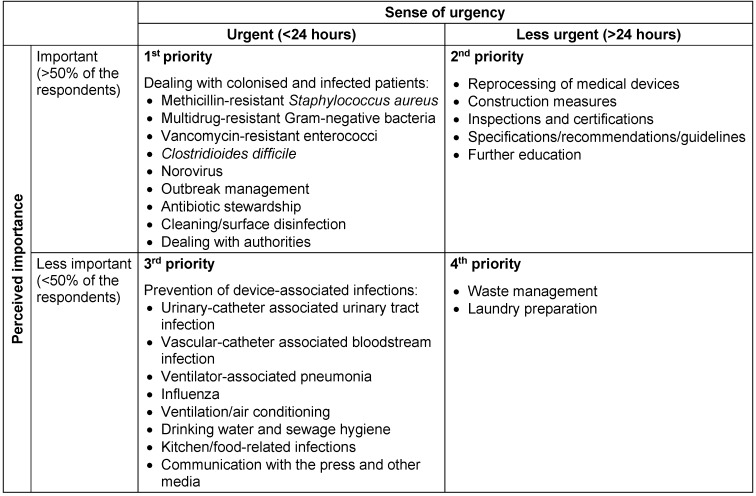
Prioritization of the consultation requests of infection control link physicians

**Table 3:**
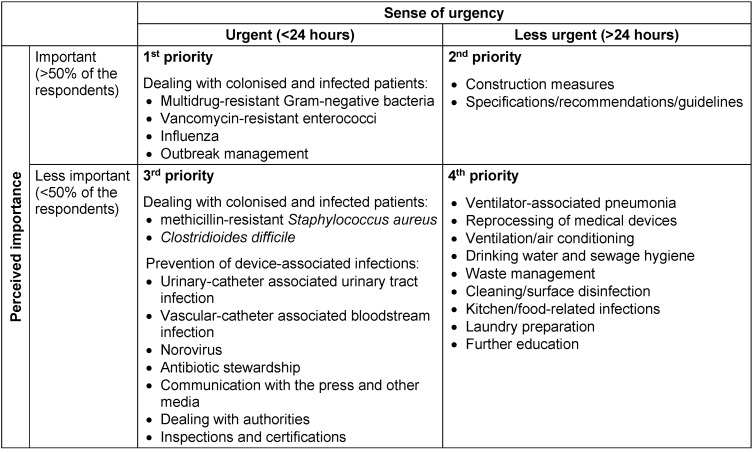
Prioritization of the consultation requests of infection control nurses

**Table 4:**
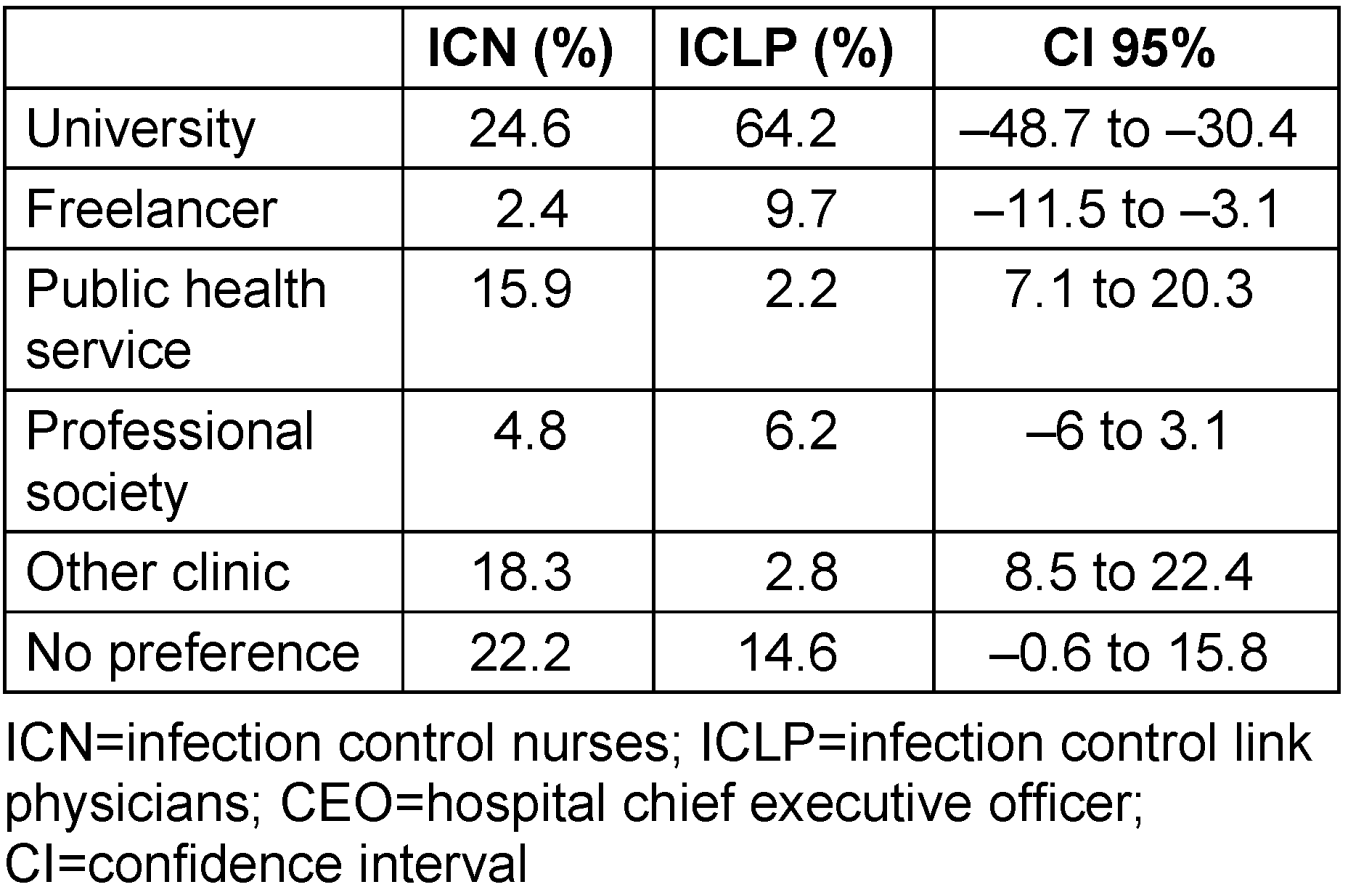
Preferred institutions for expertise and advice
